# Available Compounds With Therapeutic Potential Against COVID-19: Antimicrobial Therapies, Supportive Care, and Probable Vaccines

**DOI:** 10.3389/fphar.2020.582025

**Published:** 2020-10-06

**Authors:** Rajnish Kumar, Janmejai Kumar Srivastava, Rachana Singh, Mohammed Haris Siddiqui, Rasha A. Mansouri, Jawaher A. Abdulhakim, May N. Bin-Jumah, Saad Alkahtani, Mohamed M. Abdel-Daim, Md. Sahab Uddin

**Affiliations:** ^1^ Amity Institute of Biotechnology, Amity University Uttar Pradesh, Lucknow Campus, Lucknow, India; ^2^ Department of Bioengineering, Integral University, Lucknow, India; ^3^ Department of Biochemistry, Faculty of Sciences, King Abdulaziz University, Jeddah, Saudi Arabia; ^4^ Department of Medical Laboratory, Faculty of Applied Medical Sciences, Taibah University, Yanbu, Saudi Arabia; ^5^ Department of Biology, College of Science, Princess Nourah bint Abdulrahman University, Riyadh, Saudi Arabia; ^6^ Department of Zoology, College of Science, King Saud University, Riyadh, Saudi Arabia; ^7^ Pharmacology Department, Faculty of Veterinary Medicine, Suez Canal University, Ismailia, Egypt; ^8^ Department of Pharmacy, Southeast University, Dhaka, Bangladesh; ^9^ Pharmakon Neuroscience Research Network, Dhaka, Bangladesh

**Keywords:** antimicrobial therapy, coronavirus disease 2019, drug, severe acute respiratory syndrome coronavirus 2, supportive therapy, vaccine

## Abstract

The recent outbreak of the COVID-2019 (coronavirus disease 2019) due to the infectious severe acute respiratory syndrome coronavirus 2 (SARS-CoV-2) has realized the requirement of alternative therapeutics to mitigate and alleviate this lethal infection. These alternative therapies are effective when they are started at the initial stage of the infection. Some drugs that were used in previous other related infections SARS-CoV-2003 and Middle East respiratory syndrome coronavirus (MERS-CoV)-2012 could be potentially active against currently emerging SARS-CoV-2. This fact imparts some rationale of current interventions, in the absence of any specific therapeutics for SARS-CoV-2. It is imperative to focus on the available antimicrobial and adjunct therapies during the current emergency state and overcome the challenges associated with the absence of robust controlled studies. There is no established set of drugs to manage SARS-CoV-2 infected patients. However, closely following patients’ conditions and responding with the dosage guidelines of available drugs may significantly impact our ability to slow down the infection. Of note, it depends upon the condition of the patients and associated comorbid; therefore, the health workers need to choose the drug combinations judiciously until COVID-19 specific drug or vaccine is developed with the collective scientific rigor. In this article, we reviewed the available antimicrobial drug, supportive therapies, and probable high importance vaccines for the COVID-19 treatment.

## Introduction

Coronavirus is named after the crown-like structures projected from the envelope of its surface. Coronavirus belongs to the family Coronaviridae, which comprises of four genera: α-coronavirus, β-coronavirus, δ-coronavirus, and γ-coronavirus. α and β-coronaviruses infect mammals; however, γ and δ-coronaviruses mostly infect birds. The highly contagious coronavirus disease 2019 (COVID-19) belongs to the genus β-coronavirus (Loeffelholz and Tang, 2020; Zhu et al., 2020). It is caused by a novel β-coronavirus, severe acute respiratory syndrome coronavirus 2 (SARS-CoV-2) outbreak that has by far affected almost all the countries amounting to the death of about 850,000 individuals till date internationally.

The SARS-CoV-2 contains four structural proteins, namely, spike, membrane, envelope, and nucleocapsid (Bosch et al., 2003). The transmembrane spike proteins are trimeric glycoproteins present on the surface of the virus. These proteins are responsible for diversity and host-tropism of the virus. The virus attaches to the angiotensin-converting enzyme 2 (ACE2) (functional host-receptors for SARS-CoV-2), and through membrane fusion or endocytosis enters the host cell (Li et al., 2003; Chen Z. et al., 2020). In the alveolar space, ACE2 has highly expressed an upper side of lung epithelium cells, and therefore, SARS-CoV-2 can easily enter and abolishes these cells (Hamming et al., 2004; Jia et al., 2005; Zou et al., 2020). The released viral genetic material (single-stranded positive-sense ribonucleic acid) reaches the nucleus and starts the self-replication, transcription, and translation. The biosynthesis of viral proteins in the host-cells enables it to assemble, mature, and get released as new copies of viral particles.

The SARS-CoV-2 primarily infects respiratory system but other organs like kidney, heart, ileum, and spleen can also be infected (Kabir et al., 2020). The common symptoms of this virus infection include dyspnoea, dry cough and fever (Huang et al., 2020). However, generalized weakness, diarrhoea, vomiting, headache, and dizziness may also be observed (Shi et al., 2020). There is currently no specific therapeutics available for the prevention or cure for SARS-CoV-2 as per the Food and Drug Administration (FDA), the World Health Organization (WHO), and the Centers for Disease Control and Prevention (CDC). The WHO guidelines emphasized the role of supportive care on the basis of illness severity, i.e., symptomatic treatment for mild infections and evidence-based ventilatory management for acute respiratory distress syndrome and early recognition and treatment of bacterial infections and sepsis in severe COVID-19 cases (World Health Organization, 2020). So far, agents under clinical trials are also being used based on their *in vitro* activity against SARS-CoV-2. However, there are minimal clinical evidence could be collected so far, and the efficacy of such drug therapies in the treatment of SARS-CoV-2 has not been established. The vaccines against COVID-19 will probably take at least a year to become available, and the use of available drugs to prevent disease (chemoprophylaxis) is the main option in hand for the management of infected patients.

**Figure 1:**
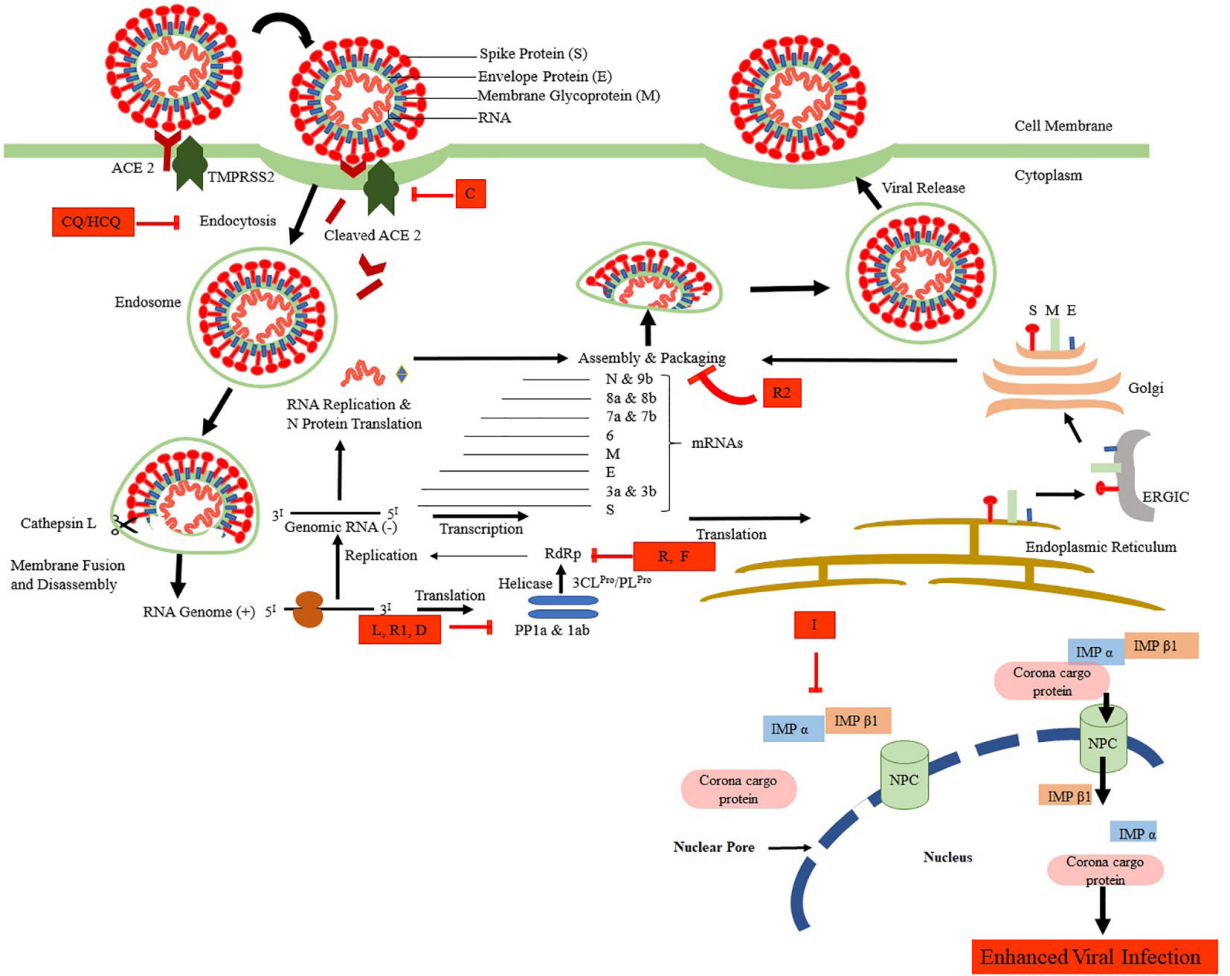
The life cycle of SARS-CoV-2 and its possible inhibitors. SARS-CoV-2 enters the target cells *via* an endosomal pathway. The S protein binds with the angiotensin-converting enzyme 2 (ACE2) of the cell primed by serine protease TMPRSS2. Viral RNA is unveiled in the cytoplasm to produce PP1a and PP1ab polyproteins, which are cleaved to form non-structural proteins. These non-structural proteins facilitate the formation of negative RNA by the process of replication and transcription. This, in turn, translates to N protein, another set of translation takes in the endoplasmic reticulum-ERGIC-Golgi to produce structural proteins (S, M, and E). Finally, the packing of viral RNA with N proteins and further assembly of S, M, and E proteins take place to form SARS-CoV-2 buds, which are released from the infected cell by exocytosis. Various drugs that are shown to inhibit the SARS-CoV-2 at various stages are CQ/HCQ, Chloroquine/Hydroxychloroquine; L, Lopinavir; R1, Ritonavir; I, Ivermectin; R, Remdesivir, R2, Resveratrol; D, Darunavir; C, Camostat.

Therefore, for the management of the SARS-CoV-2, the current treatments are entirely supportive by nature. In general, pharmacological treatment is not suggested for young patients with mild indications and without other underlying chronic conditions (Gao et al., 2020). **Figure 1** shows the life cycle of SARS-CoV-2 along with its possible inhibitors at various stages of its attachment, multiplication, and growth in the host cell. Available antimicrobials, adjunctive, supportive therapies, and probable vaccines are discussed in the following sections. Dugs and support therapies that are currently under clinical are listed in the **Supplementary Material**.

## Antimicrobial Therapies Against COVID-19

### Chloroquine

**Figure 2:**
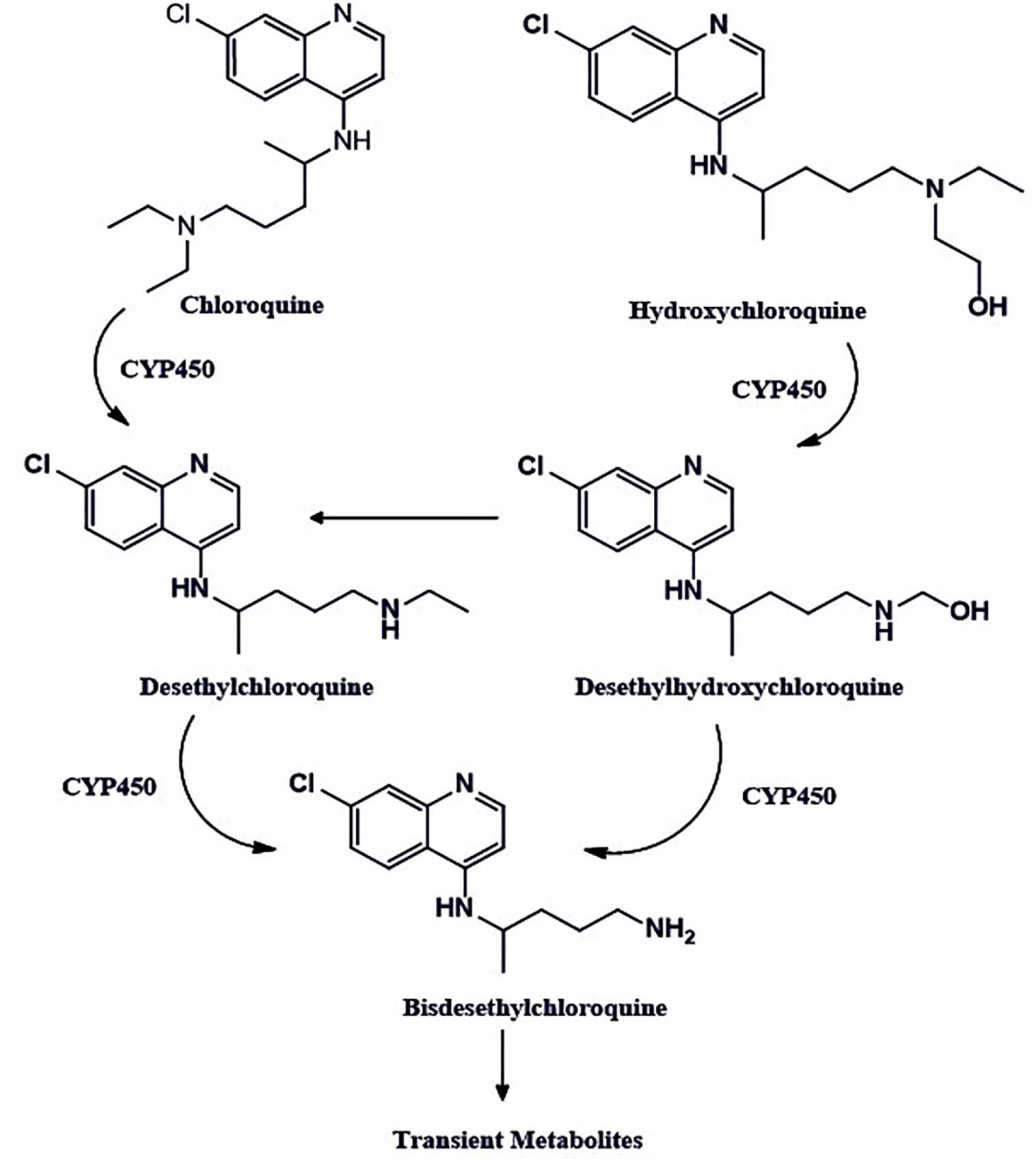
The chemical structures of chloroquine and hydroxychloroquine. The chloroquine contains the N-diethyl group, whereas hydroxychloroquine has an N-hydroxyethyl group at the same position, which is the only structural difference these two drugs have. Both drugs share bis-desethylchloroquine as a common metabolite.

Chloroquine (**Figure 2**) has been used for years against malaria. This cost-effective and widely available therapeutic agent is also a robust antiviral, as it blocks a virus from invading the human cells. A large number of research groups are currently reviewing whether it effectively decreases the viral load in patients with SARS-CoV-2 (Savarino et al., 2006; Marmor et al., 2011; Cortegiani et al., 2020). However, *in vitro* and limited clinical data indicate therapeutic advantages against SARS-CoV-2 infection. SARS-CoV-2 is an enveloped virus, and it enters the cell by endocytosis. Endocytosis is a cellular process in which elements are taken into the cell by making a tiny vesicle. Once inside, a dip in pH promotes the fusion of the virus envelope with the membrane of the vesicle that encompasses it, to be released into the cytoplasm (McChesney, 1983). Till date, chloroquine is verified effective against COVID-19 *in vitro* through influencing bis (monoacylglycero) phosphate entry by controlling the endocytic pathway (Carrière et al., 2020). The use of chloroquine against COVID-19 might be a risk and require continuous monitoring until confirmed clinical evidence are available.

### Hydroxychloroquine

Hydroxychloroquine is also classified under the antimalarial agent. It is a comparatively less toxic analog of chloroquine, which has already been found to stop the replication of SARS-CoV-2 *in vitro* in cell culture studies (Colson et al., 2020; Yao X. et al., 2020). It exhibits the mechanism of action, similar to the chloroquine for COVID-19 patients. It primarily increases the pH in the endosomes, thereby, stops their acidification and maturation and interferes with the entry of the virus in the cell (Hacker et al., 1998; Macfarlane et al., 1998; Vincent et al., 2005; Christophe et al., 2006). Also, it inhibits the toll like receptors (TLRs) and thereby reduces the pro-inflammatory cytokines Interleukin-6 production (Fox, 1993; Fox, 1996; Ben-Zvi et al., 2012; Jorge et al., 2018; Sahraei et al., 2020; Wu et al., 2020a).

Hydroxychloroquine is being used as a potential therapeutic agent for the treatment of SARS-CoV-2 infected patients under the FDA-Emergency Use Authorization (EUA) by following the same guidelines as recommended for chloroquine. However, it is not an FDA-approved drug for the treatment of SARS-CoV-2 infection. Hydroxychloroquine shows better *in vitro* efficacy against SARS-CoV-2 as compared to the chloroquine. Effective concentration-50 (EC50) values of the hydroxychloroquine were reported to be 6.25 µM at 24 h and 5.85 µM at 48 h as compared to chloroquine, which is about 100 µM at 24 h and 18.01 µM at 48 h. Moreover, hydroxychloroquine has comparatively better compatibility with other antiviral agents e.g., lopinavir, ritonavir, ribavirin, oseltamivir, remdesivir, etc., which could be used concomitantly during COVID-19 treatment (Wang et al., 2020). In addition, it does not interfere with intravenous immunoglobulins and interferons. Although there are some common adverse drug reactions of hydroxychloroquine such as nausea, stomach pain, vomiting, headache, and in some cases, itching, they do not require discontinuation of the drug.

Prolonged use of the drug may lead to some significant side effects irregular heartbeats, yellowing of the eyes, convulsions or seizures, retinopathy, blurred vision, muscle weakness, difficulty hearing, bruising, or bleeding of the skin, etc. (Ling Ngan Wong et al., 2008; Marmor et al., 2011). Therefore, risk and benefit balance assessment must be done while recommending the hydroxychloroquine in such cases. There are *in vitro* and clinical results in support (Gautret et al., 2020; Yu et al., 2020) and against (Cavalcanti et al., 2020; Chen J. et al., 2020; Geleris et al., 2020; Molina et al., 2020; Rosenberg et al., 2020) the use of hydroxychloroquine for COVID-19 patients. Of note, the data available so far suggest that therapeutic efficacy of HCQ in COVID-19 is well overshadowed by its ineffectiveness and detrimental effects as shown by some of the above-referred studies. Therefore, it is suggested to avoid use of HCQ for COVID-19 patients (unless necessary) until clear, sufficient, well-conducted, randomized, and controlled clinical data is available to reach the right conclusions.

### Lopinavir and Ritonavir

**Figure 3:**
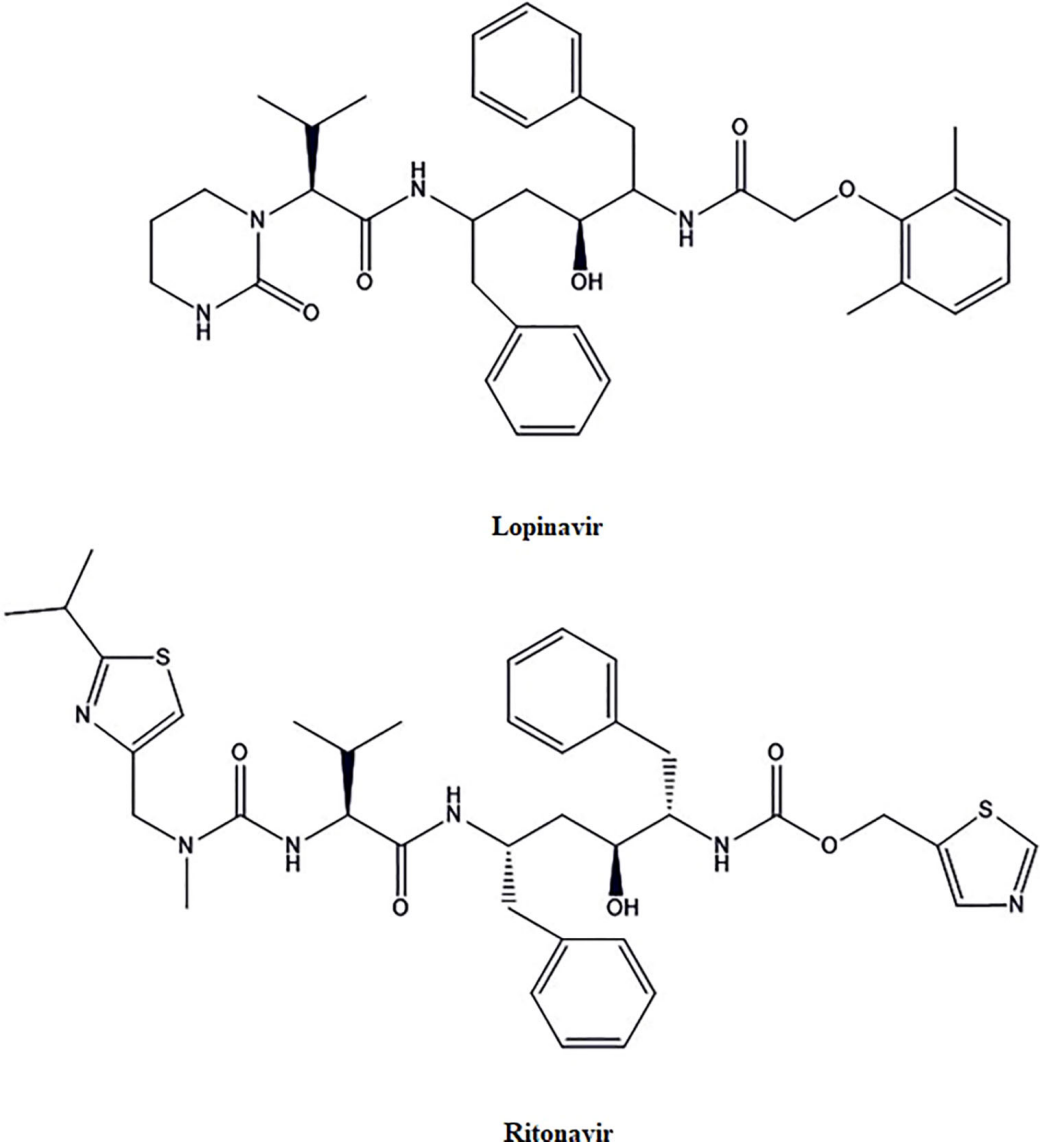
The chemical structures of lopinavir structure of ritonavir.

Lopinavir and Ritonavir (**Figure 3**) are HIV Protease inhibitors. Animal model and in- vitro studies suggest that lopinavir and ritonavir have therapeutic activity for SARS-CoV and Middle East respiratory syndrome coronavirus (MERS-CoV), but their role in the treatment of SARS-CoV-2 is not clear (Chen et al., 2004; Yao T. T. et al., 2020). These drugs may bind to the Mpro, which is a vital enzyme for the SARS-CoV-2 replication. Mpro facilitates proteolytic processing, which is released by auto-cleavage of pp1a and pp1ab. Then Mpro, in turn, cleaves pp1a and pp1ab to release functional proteins necessary for viral replication (Adedeji et al., 2014; Liu et al., 2020). A retrospective cohort study was done to review the clinical course, and risk factors for mortality during lopinavir-ritonavir therapy of the SARS-CoV-2 infected hospitalized patients (n=29). There was no difference found in the virus shedding duration with lopinavir-ritonavir (Zhou F. et al., 2020).

As per another recent randomized, open-label, controlled trial (n=199) studies, lopinavir-ritonavir treatment applied to standard supportive care did not result in clinical improvement or mortality in severely infected patients with SARS-CoV-2 as compared to the patients on standard supportive care only. However, the median time to the clinical improvement was found to be reduced by one day with lopinavir-ritonavir treatment (Cao et al., 2020). This modest improvement still seems to be significant because the study was carried out on the severely infected patients with a mortality rate of 22.1% as compared to the 11%–14.5% as reported in preliminary descriptive studies of hospitalized SARS-CoV-2 infected patients (Chaolin et al., 2020; Chen N. et al., 2020; Osborne et al., 2020). After analyzing the recently published clinical outcomes, it was found that the clinical improvement time was not significantly reduced for SARS-CoV-2 infected patients upon the use of lopinavir-ritonavir as compared to standard care. The study by Cao et al. (2020) demonstrating lower acute respiratory distress syndrome (ARDS) with lopinavir-ritonavir and less adverse drug reactions as compared to the standard care. Overall, the effectiveness of lopinavir-ritonavir in severe SARS-CoV-2 could not be considered very encouraging until more conclusive clinical data is available.

Further, trials with the optimal sampling methods and specimen from the lower respiratory tract from the mild to severe SARS-CoV-2 infected patients may lead to better conclusions for lopinavir-ritonavir treatment. More clinical trials (NCT04261907, NCT04321174, NCT04330690, ChiCTR2000030187) data for evaluating the therapeutic efficacy of lopinavir-ritonavir in COVID-19 are awaited.

### Ivermectin

**Figure 4:**
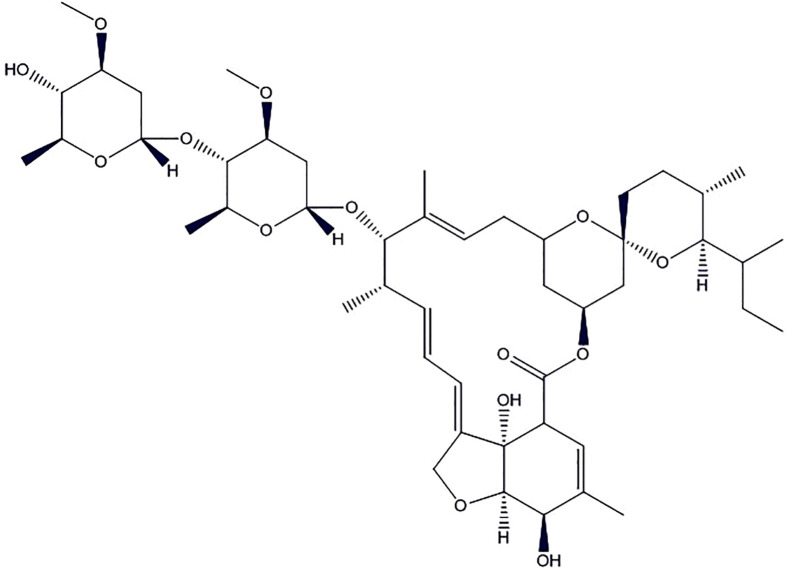
The chemical structure of ivermectin.

Ivermectin (**Figure 4**) is a broad-spectrum antiparasitic drug but shown promises of antiviral activity in recent *in vitro* studies (Canga et al., 2008; Wagstaff et al., 2012; Lundberg et al., 2013; Tay et al., 2013; Götz et al., 2016). Importin (IMP) α/β1 is a heterodimer which facilitates the nuclear import of coronavirus cargo protein, present in the cytoplasm of the infected cell. It translocates coronavirus protein through the nuclear pore complex (NPC) inside the nucleus where the complex gets separated. The cargo could diminish the antiviral response of the infected cell, leading to increased infection. Ivermectin binds to Impα/β1 heterodimer, thereby destabilize and prevents Impα/β1 from interacting with viral protein. It leads to the stoppage of the translocation of the viral protein into the nucleus. This mechanism is further substantiated by various studies to demonstrate the effect of ivermectin on RNA-virus infections, e.g., human immunodeficiency virus-1 (HIV-1) (Wagstaff et al., 2011), Venezuelan equine encephalitis virus (VEEV), influenza, West Nile Virus (Yang et al., 2020), and Dengue virus (DENV) 1-4. This is due to the dependence of these RNA viruses on IMPα/β1 during infection (Caly et al., 2012; Jans et al., 2019).

Of note, the FDA has only approved this drug for the treatment of parasitic worms such as onchocerciasis and intestinal strongyloidiasis along with some topical formulations for the human to be used upon prescription only for external parasite treatment such as rosacea and head lice. One of the recent *in vitro* studies conducted by Clay et al. (2020) confirmed the antiviral activity of ivermectin against SARS-CoV-2. The SARS-CoV-2 infected Vero/hSLAM cells were subjected to 5 µM ivermectin for 48 h. The study indicates about 5,000-fold decrease in viral RNA as compared to the control. This was the first study to assess and demonstrate the effectiveness of ivermectin in COVID-19. The authors reported that the ivermectin may have antiviral effects by inhibition of IMP α/β receptor, which is responsible for transmission of the viral proteins into the nucleus of host cell. Based on the findings in the study authors proposed clinical studies to further confirm the potential benefits of this drug in the treatment of COVID-19 (Heidary and Gharebaghi, 2020). It is worth mentioning here that ivermectin activity in cell has not been reproduced in the mouse infection models for many viruses. Also, its activity has been not yet proven clinically. The concentration of drug required to be effective in SARS-CoV-2 infected cell culture is in the microgram range but its safe blood levels for imparting therapeutic activity is 20–80 ng/ml. Still, the ivermectin is being available worldwide which may be due to its favorable pharmacokinetics and therapeutic window. Therefore, treatment with ivermectin for COVID-19 patients should be strictly by licensed health workers after a careful review of the patient’s health condition until robust and conclusive clinical data (NCT04360356, NCT04343092, NCT04351347) are available.

### Remdesivir

**Figure 5:**
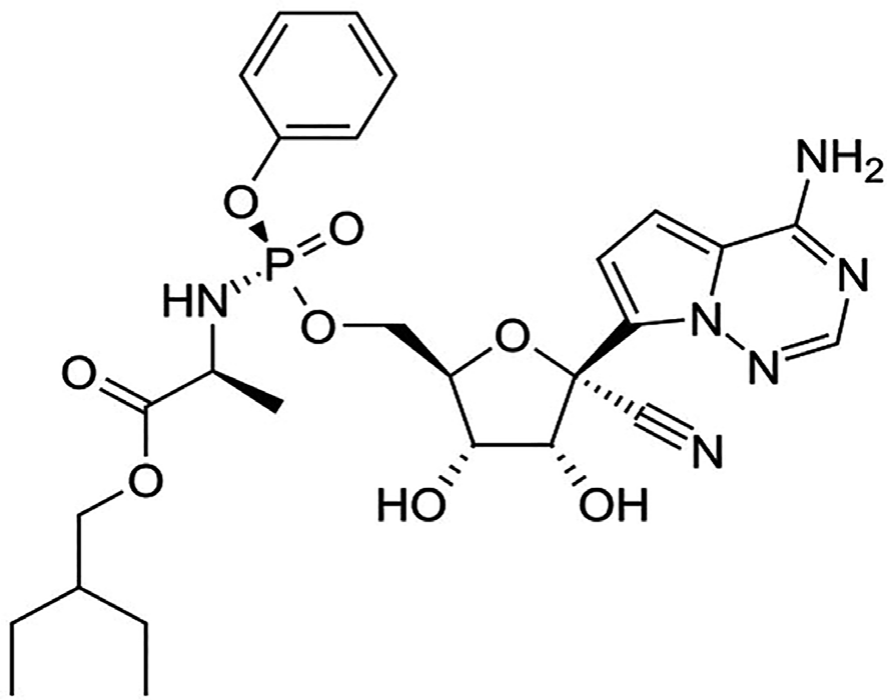
The chemical structure of remdesivir.

Remdesivir is an investigational nucleoside analog and available only through compassionate use and study protocols. The *in vitro* studies suggest that it exhibits antiviral activity including SARS-CoV-2 (Agostini et al., 2018; Brown et al., 2019; de Wit et al., 2020; Ko et al., 2020; Gordon et al., 2020). Mono-phosphoramidate remdesivir is a prodrug of remdesivir-triphosphate (Warren et al., 2016). Remdesivir (**Figure 5**) is a 1′-cyano-substituted adenosine nucleotide analog that undergoes a metabolic mechanism, activating nucleoside triphosphate metabolite, which inhibits the RNA dependent RNA polymerase (RdRp).

Remdesivir-triphosphate competes with adenosine-triphosphate (ATP) and gets incorporated in the viral RNA. The RNA synthesis terminates after three nucleotides are further added from the position of remdesivir-triphosphate incorporation (Sheahan et al., 2017). The *in vitro* studies suggest that remdesivir exhibits potential therapeutic activity against clinical isolate [Half-cytotoxic concentration (CC50) > 100 mcgM; EC50 = 0.77 mcgM; Selective index (SI) > 129.87] of SARS-CoV-2. One of the recent studies reported that the clinical improvements were observed in 68% (36 out of 53 patients) of the hospitalized COVID-19 patients when treated with remdesivir on a companionate use basis (Grein et al., 2020). However, currently reported studies have some vital limitations such as the small sample size, the relatively less duration of follow-up, potential missing data, the insufficient of information on some patients that were initially involved in the study, and the absence of a randomized control group. Currently, several clinical trials for the evaluation of the efficacy of remdesivir in COVID-19 patients is being conducted (NCT04302766, NCT04323761). More significant and conclusive clinical evidence is expected in the coming months.

### Favipiravir

**Figure 6:**
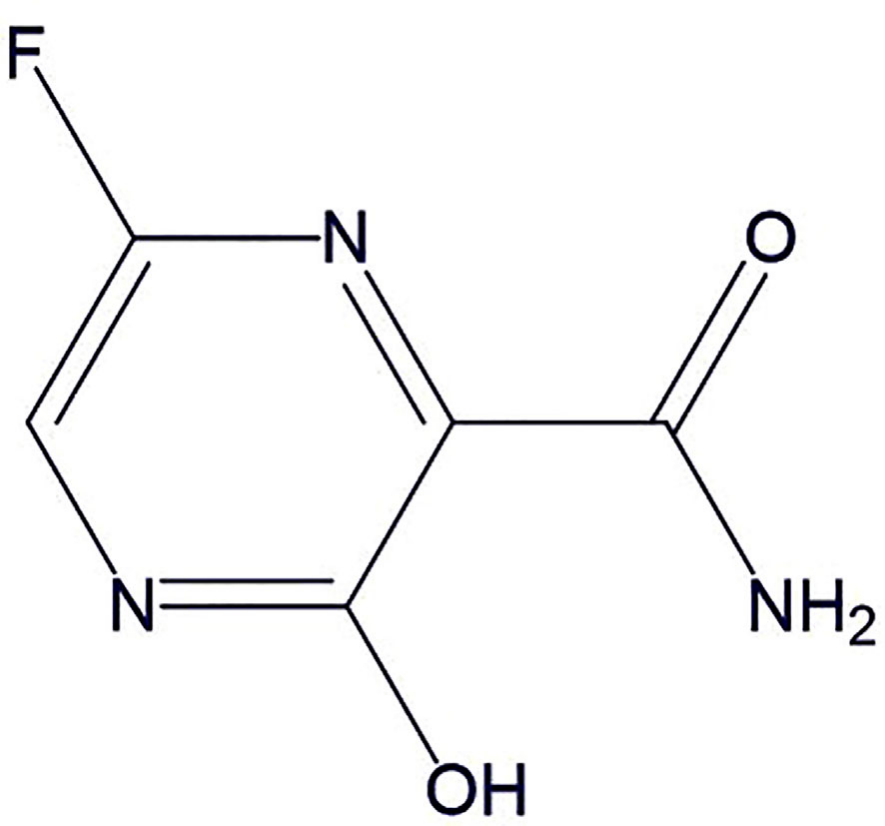
The chemical structure of favipiravir.

Favipiravir (**Figure 6**) inhibits RNA-dependent RNA polymerase (RdRp). It structurally resembles the endogenous guanine and shows broad-spectrum antiviral activity through competitive inhibition (Furuta et al., 2013). The *in vitro* studies suggest its therapeutic activity against RNA viruses (respiratory syncytial virus, rhinovirus, and, poliovirus) (Furuta et al., 2002; Dong et al., 2020; Shiraki and Daikouku, 2020; Yanai, 2020). Recently, Wang et al. (2020) conducted *in vitro* and demonstrated the effectiveness of favipiravir against SARS-CoV-2. In another open-label control study was done by Cai et al. (2020) to assess therapeutic potentials of favipiravir against SARS-CoV-2. Total of 35 patients were given oral favipiravir with interferon-α aerosol inhalation and a significant reduction in viral clearance time was observed. The multivariable Cox regression analysis further confirmed that the favipiravir was individually associated with faster clearance of the virus. The favipiravir also demonstrated noteworthy improvement in chest imaging and significantly smaller adverse reactions (e.g., nausea, vomiting, etc.) as compared to the lopinavir/ritonavir treated patients. Further, case reports for effectiveness of favipiravir are also reported by the Japanese association for infectious diseases on their web symposium as it was able to improve the clinical symptoms by 90%, 85%, and 61%, in mild, moderate, and severe COVID-19 cases, respectively after 14 days from the start of the treatment (Japanese Association for Infectious Diseases, 2020) Based on the evidence, favipiravir may be a relatively safe and effective drug for COVID-19 at present and expected to emerge as a broad-spectrum antiviral drug against SARS-CoV-2. Its efficacy (NCT04349241, NCT04358549, NCT04346628, NCT04351295, ChiCTR2000030113) and different combinations with other drugs (favipiravir + hydroxychloroquine: NCT04359615; favipiravir + tocilizumab: NCT04310228; favipiravir + imatinib + telmisartan + hydroxychloroquine: NCT04356495) are being clinically evaluated for effectiveness against COVID-19.

Apart from the above-mentioned antimicrobial agents, there are some drugs such as darunavir, oseltamivir, nitazoxanide, and ribavirin, that are being discussed in the scientific community to have efficacy against SARS-CoV-2. However, due to the absence of clinical evidence or higher risk factors in comparison to the benefits, such drugs are not recommended to be used in COVID-19. Darunavir is an HIV protease inhibitor that is being considered for its therapeutic activity against COVID-19; however, there are no *in vivo* or clinical data available so far, to support the pharmacological efficacy and safety of the drug for COVID-19 patients. The oseltamivir is a neuraminidase inhibitor SARS-CoV-2 does not utilize the neuraminidase enzyme for their budding and multiplication (McKimm-Breschkin, 2012). Therefore, no therapeutic activity is expected by oseltamivir for COVID-19. Nitazoxanide shows some activity against SARS-CoV-2. However, it does not demonstrate any benefit when tested clinically (Gamino-Arroyo et al., 2019).

Moreover, its safety profile and tolerance are also some of the major concerns. Another antiviral agent ribavirin is a guanosine analog that inhibits the replication of DNA and RNA virus. It has shown mixed pharmacological efficacy toward other novel viruses, e.g., SARS-CoV and MERS‐CoV (Graci et al., 2006). However, there is no conclusive clinical demonstration of its efficiency toward SARS-CoV-2. Also, the dosage used for its testing for MERS-CoV (800-3600mg/Day) poses a significant adverse reaction profile (Gross and Bryson, 2015; Mo et al., 2016; Arabi et al., 2019). Though, outcomes of some clinical trials for ribavirin in combination with other drugs (ribavirin + lopinavir/ritonavir + interferon‐β1b are: NCT04276688, ribavirin+ interferon (IFN)-α/lopinavir-ritonavir/chloroquine phosphate/arbido: NCT04306497) are still awaited.

## Adjunctive/Supportive Care Against COVID-19

### Monoclonal Antibodies

#### Tocilizumab

It is widely reported that the SARS-CoV-2 activates the innate immunity and the levels of numerous cytokines, such as interleukin (IL)-1β, IL-6, IL-8, TNF, granulocyte-macrophage colony-stimulating factor (GM-CSF), and granulocyte colony-stimulating factor (G-CSF), along with chemokines, such as monocyte chemoattractant protein (MCP)1, macrophage inflammatory protein (MIP)1α, and interferon gamma-induced protein (IP10) get increased with higher levels in those who are severely infected (Schett et al., 2020; Zhang C. et al., 2020; Zhang W. et al., 2020). Tocilizumab competitively inhibits the soluble as well as membrane-bound IL-6 receptor and thereby interferes with its diverse mediated signaling process. IL-6 is formed by T-cells, B-cells, monocytes, lymphocytes, and fibroblasts. This pro-inflammatory cytokine, which is associated with various physiological processes, e.g., activation of T-cell and immunoglobulin secretion, acute-phase protein synthesis initiation in the liver, and activation of hematopoietic precursor cell differentiation and proliferation. Tocilizumab is given intravenously in the treatment of COVID-19 in some countries such as China, and Italy resulted in encouraging outcomes (Harrison, 2020). In one of the retrospective reviews, 21 SARS-CoV-2 infected patients were analyzed, when tocilizumab was added to the standard therapy. Clinical symptoms, lymphocyte percentage, computed tomography (CT) opacity changes, and C‐reactive protein (CRP) levels were reported to be improved in these patients, which suggests that tocilizumab has clinical benefits (Xu et al., 2020). However, there were no comparators reported in this study.

In another retrospective study, laboratory descriptors of IL‐6 and CRP were assessed for prior and after tocilizumab treatment of 15 COVID-19 patients (7 critically ill; 6 seriously ill; 2 moderately ill). The CRP levels rapidly ameliorated in all the patients, and the IL‐6 level in serum was found to further increased initially and then reduced [decreased from 126.9 (10.7–257.9) to 11.2 (0.02–113.7) mg/L (P < .01)] after tocilizumab therapy in 10 patients. However, 3 patients who received only one dose of the tocilizumab failed to recover (died), and 2 showed disease aggravation (Luo et al., 2020). Although the above-mentioned studies reported a better response of tocilizumab in COVID-19 patients, one should be cautious while evaluating the outcomes as the reported cases and the duration of treatment (ranging from 2–7 days) still seems to be insufficient. Furthermore, the use of laboratory parameters to express disease activity is a difficult task and may influence the outcome of the study. More significant clinical data for the efficacy of tocilizumab in the management of COVID-19 is awaited from ongoing trials (NCT04335071, NCT04356937, NCT04363853, NCT04317092, etc.).

#### Sarilumab

Sarilumab is also an IL-6 receptor inhibitor that interferes with the IL-6 driven through binding with both membrane-bound and soluble IL-6 (sIL-6R and mIL-6R) receptors. The clinical efficacy of sarilumab for COVID-19 patients is under the process of evaluation (NCT04324073; NCT04315298; NCT04327388; NCT04322773; NCT04321993), and the pharmacological potential of it could only be concluded once the robust data evidence are available (Menzella et al., 2020).

#### Leronlimab

Leronlimab is an investigational humanoid monoclonal antibody and may be used for mitigation of the cytokine storm, which could be a major component in severely diseased COVID-19 patients. It binds with chemokine receptor-5 (CCR5) to alleviate the cytokine release syndrome. The evaluation of leronlimab is now being done in a limited number of severely SARS-CoV-2 infected patients with the emergency investigational new drug application, FDA (CytoDyn, 2020). Data from ongoing clinical trials (NCT04343651, NCT04347239) for the evaluation of therapeutic efficacy and safety of leronlimab will yield a better understanding of its role in the management of COVID-19.

### Baricitinib

Baricitinib is a Janus kinase (JAK) inhibitor. Cytokine receptor interactions with cell-membrane produce signals which are further transmitted by the intracellular JAK enzymes to impact the process of immune cell function and hematopoiesis. JAKs phosphorylate and trigger signal transducers and activators of transcription proteins (STATs) during this signal transmission pathway, which affects the intracellular activity. Baricitinib modulates the signaling pathway by preventing phosphorylation of JAKs and thereby inhibiting the STATs activation. The cytokine signal transduction takes place by the pairing of JAKs. Baricitinib has a higher binding affinity for JAK1, JAK2, and tyrosine-protein kinase-2 (TYK2), as compared to JAK3. It inhibits the cytokine activated STAT phosphorylation facilitated by JAKs (JAK1-JAK2, JAK1-JAK3, JAK1-TYK2, or JAK2-TYK2) in the human leukocytes. Baricitinib is an approved drug for rheumatoid arthritis therapy, and if it could pose an antiviral activity by inhibiting JAK-STAT signaling with the same approved dose, that would be a great advantage. However, at the time of early viral infection JAK-STAT signaling pathway (primarily JAK1 and JAK2) activated by interferon gives rise to the increase in the regulation of several interferon-controlled genes that rapidly kill viruses in infected cells (Favalli et al., 2020). To crack this mechanism, many viruses, have developed viral encoded factors to counteract and antagonize the JAK-STAT pathway, which is a crucial descriptor of their viral effect (Fleming, 2016). Therefore, inhibiting the JAK-STAT signaling by baricitinib seems to be substantial facilitation for viral evolution in the host cell.

This argument poses an interesting question upon the general use of baricitinib as an adjunct therapy for COVID-19 patients. However, it is also speculated that patients with the early asymptomatic infection do not need hospitalization, and about 80% of patients can get cured, mostly by endogenous antiviral mechanisms, which certainly includes the interferons (Richardson et al., 2020; Stebbing et al., 2020). Such patients do not require JAK-STAT signaling inhibition. Only the hospitalized patients with after the peak SARS-CoV-2 load (after about 7–10 days of indication onset) show the hyper inflammation due to the so-called cytokine storm resulted from a severe infection, may require the baricitinib therapy. Of note, this clinically critical stage is supported by high level signaling through JAK-STAT pathway, and thereby, enhanced interferons α+β and IL-6 levels are reported in the patients. In one of the recent pilot study, 12 moderately SARS-CoV-2 infected patients were found to show improvements in clinical and laboratory parameters with baritinicib (Cantini et al., 2020). In another non-controlled, retrospective cohort study with 15 moderate to severe COVID-19 patients, it was reported that baritinicib with HCQ leads to recovery and clinical improvements (Titanji et al., 2020). However, the efficacy of baritinicib could not be confirmed with scant and insufficient clinical results until enough data about the clinical efficiency and safety profile of baricitinib (NCT04321993, NCT04320277) is available.

### COVID-19 Convalescent Plasma

The rationale behind this therapy is that the patient recovered from the COVID-19 may contain antibodies against SARS-CoV-2 in their plasma. Though convalescent plasma (CP) is not intended to prevent COVID-19, it may be used for the severely ill patients at the life-threatening stages as the last resort to improve the survival rate. Of course, the clinical evaluation data for its efficacy must be substantial to prove its effectiveness against the virus. There is evidence that CP works well in reducing the mortality rate of severely infected patients without the occurrence of severe adverse events (Soo et al., 2004; Cheng et al., 2005; Lai, 2005; Chen L. et al., 2020). Moreover, such treatment has worked well with MERS (Arabi et al., 2015) influenza A H1N1 pandemic 2009 virus (Hung et al., 2013), and HIV 1 (Lu et al., 2016). In a timely descriptive study, the effects of ABO-compatible CP therapy were evaluated on 6 COVID-19 positive patients. The evaluation was done based on symptoms improvement, radiologic changes, and laboratory testing. The therapy resulted in the resolution of GGOs and the consolidation of 5 out of 6 patients. There was an instant rise in the anti-SARS-CoV-2 antibody titers in 2 patients, but not all (Ye et al., 2020). These findings suggest that PC therapy is not only effective but also specific in COVID-19. In another CP evaluation study, 6 COVID-19 positive patients (tested by IgM ELISA) were given the therapy. After 11 days of CP transfusion, these patients did not need ventilator support (Zhang L. et al., 2020), which is indicative of the positive response of CP in COVID-19 treatment.

In another study, 5 severely ill patients with ARDS were given CP (two successive dosages of 200–250 ml) along with SARS-CoV-2-specific immunoglobulin G (IgG) titer. Four out of five patients showed body temperature reduction just after 3 days and an increase in the partial pressure of arterial oxygen and percentage of inspired oxygen ratio (PAO_2_/FIO_2_) after 12 days of treatment (Shen et al., 2020). Their sequential organ failure assessment score also reported being decreased along with the viral load, which became normal after 12 days of CP transfusion. After 14 days of the treatment, mechanical ventilation was not required, which is further indicative of the recovery of the COVID-19 patients. The investigational new drug (IND) regulatory steps led down by FDA to be followed by the investigators/licensed physicians to participate in the study who may have collected the CP from the COVID-19 recovered patients. CP seems to be an effective and safe therapy for critically ill patients infected with SARS-CoV-2. However, some clinical outcomes from multicentered randomized controlled trials are required to substantiate therapeutic promises of CP. A large number of clinical trials are going on for assessment of therapeutic potency and safety of CP in COVID-19, and most of them are currently in phase 2 (NCT04359810, NCT04347681, NCT04345991, NCT04343261).

### Conditional Support Therapy

#### Bronchodilators

Bronchodilators have the potential to enhance the risk of viral transmission with nebulized therapy. Therefore, there is not much role of bronchodilators in the supportive therapy and management of COVID-19 patients except there are chronic obstructive pulmonary disease or asthma (Institute for Safe Medication Practices, 2020). Even with such underlaying diseases, just the metered dose of inhalers is recommended.

#### NSAIDs

The role of nonsteroidal anti-inflammatory drugs (NSAIDs) in the management of COVID-19 patients is still under investigation (FDA, 2020). Drugs such as ibuprofen may increase angiotensin-converting enzyme 2 (ACE2) expression in the diabatic that could lead to detrimental results in COVID-19 patients. Ibuprofen may also increase the ACE2 expression in case of angiotensin II type-I receptor blockers treated patients (Loutfy et al., 2003; Fang et al., 2020). As a result, it is also suggested that the enhanced expression of ACE2 in the comorbid patients may facilitate the COVID-19 infection. Though, acetaminophen may be carefully used for fever control in the critically ill COVID-19 patients with high temperatures.

#### Corticosteroids

Corticosteroids (prednisone, methylprednisolone, dexamethasone, and budesonide) may be considered for patients with ARDS or refractory shock (Lian et al., 2020; Wu et al., 2020b; Zhou Y. H. et al., 2020). After exploring the published literature for corticosteroids against coronaviruses, we found that more studies are exploring the therapeutic promises of corticosteroids rather than NSAIDs (Lam et al., 2004; Wong et al., 2004; Chihrin et al., 2005). Overall, corticosteroids were found to have a positive response with respect to SARS-CoV due to their known capability to modulate the inflammations and reduce the immunopathological injury. One of the studies with porcine respiratory coronavirus infected pigs suggests that dexamethasone treatment with one or two doses of corticosteroids lessen early proinflammatory response. However, the prolonged use of corticosteroids may enhance the replication of virus (Zhang et al., 2008). On the contrary, a separate study with SARS-CoV patients indicated that early high dose of steroids with a quinolone gives the most favorable patient outcomes (Zhao et al., 2003). The studies on SARS-CoV infected mice model when treated with dexamethasone showed the alleviation of pulmonary inflammation (Hao et al., 2005).

Recent preliminary clinical trial results suggest that dexamethasone is effective in the life-saving of critically ill COVID-19 patients. This randomized, open-label controlled, adaptive, platform trial showed that dexamethasone reduced the mortality by one third and one fifth in patients getting invasive mechanical ventilation and oxygen support without mechanical ventilation, respectively. However, dexamethasone dose was not effective in reducing the mortality rate in patients not receiving any respiratory support (RECOVERY Collaborative Group, 2020). Results of the clinical trials for various corticosteroids, e.g., prednisone: NCT04344288, NCT04359511; methylprednisolone: NCT04343729; dexamethasone: NCT04360876; inhaled budesonide: NCT04355637 are waited for more clear evidence for the efficacy of corticosteroids in COVID-19.

#### Inhaled Pulmonary Vasodilators

The data about the use of pulmonary vasodilators are under investigation and aerosolized vasodilators to be avoided in the management of COVID-19 patients. There is no evidence to date about the efficacy of inhaled pulmonary vasodilators such as prostacyclins, nitric oxide, phosphodiesterase inhibitors, endothelin receptor antagonists, etc. in acute respiratory failures in SARS-CoV-2 infection (American Association for Respiratory Care, 2020; Department of Defense, 2020). Data regarding the clinical efficiency of nitric oxide are under investigation (NCT04305457, NCT04306393).

#### Anticoagulation

Low molecular weight heparin may be recommended to the COVID-19 patents, in the absence of contraindications such as platelet count < 25 x 109/L or active bleeding for venous thromboembolism prophylaxis. Therapeutic intensity anticoagulants may not be recommended for COVID-19 if venous thromboembolism is not present. During the heparin treatment with 449 COVID-19 patients, an increased D-dimer has been reported in the infected hospitalized patients. Mortality of the heparin treated patients was reported to be lower than non-treated patients after 28 days with SIC score ≥ 4 (Tang et al., 2020; Thachil et al., 2020). Ongoing clinical trials (NCT04345848, NCT04362085, NCT04359277, NCT04367831) data for therapeutic and prophylactic efficacy of anticoagulation in COVID-19 will establish better conclusions.

## Probable Vaccines

A total of 31 possible vaccines against COVID-19 have already reached to the clinical trials with 142 in the pre-clinical trials (**Table 1**). One such probable vaccine in the clinical trial stage is the recombinant adenovirus vector type 5-based vaccine, developed from the SARS-CoV-2 S gene using a non-replicating viral vector platform (Phase 1- ChiCTR2000030906; Phase 2-ChiCTR2000031781, NCT04313127). This study is sponsored by CanSino Biologics Inc. in Hubei, China and to be manufactured by Beijing Institute of Biotechnology, China. Phase 1 (n=108 healthy individuals) of the vaccine trial is an open, dose-escalating, and single-center study to assess the adverse reactions after 7 days of vaccine administration. Phase 2 (n= 500 healthy individuals) is randomized, placebo-controlled, and double-blind to assess the immunogenicity and safety by finding out the adverse reaction within 0-14 days, anti-SARS-CoV-2 antibody and SARS-CoV-2 neutralizing response after 28 days of the vaccine administration using intramuscular route.

**Table 1:** Probable vaccines under clinical trials for COVID-19 (as of August 28, 2020).

Vaccine Platform	Candidate Vaccine	Manufacturer/Developer	Route of Administration	
			Intramuscular	Intradermal
RNA	LNP-encapsulated mRNA	NIAID/Moderna	•	
	3 LNP-mRNAs	Pfizer/Fosun Pharma/BioNTech	•	
	mRNA	Curevac	•	
		Duke-NUS/Arcturus	•	
		Walvax Biotech/Academy of Military Sciences (PLA)	•	
	LNP-nCoVsaRNA	Imperial College, London	•	
Protein subunit	Adjuvanted recombinant protein (RBD-Dimer)	Chinese Academy of Sciences (Microbiology Institute)/Anhui Zhifei Longcom Biopharmaceutical	•	
	RBD-based	Kentucky Bioprocessing, Inc	•	
	Adjuvanted recombinant SARS CoV2 glycoprotein nanoparticle vaccine	Novavax	•	
	Trimeric subunit Spike protein (Native) vaccine	GSK/Dynavax/Clover Biopharmaceuticals	•	
	Advax™ adjuvant + Recombinant spike protein	Vaxine Pty Ltd/Medytox	•	
	Molecular clamp stabilized spike protein with MF59 adjuvant	CSL/Seqirus/University of Queensland	•	
	S-2P protein + CpG 1018	NIAID/Dynavax/Medigen Vaccine Biologics Corporation	•	
	RBD + Adjuvant	Instituto Finlay de Vacunas, Cuba	•	
	Peptide	FBRI SRC VB VECTOR, Rospotrebnadzor, Koltsovo	•	
Non-replicatingviral vector	Adenovirus type 5 vector	CanSino Biological Inc./Beijing Institute of Biotechnology	•	
	Ad26COVS1	Janssen Pharmaceuticals	•	
	Adeno-based	Gamaleya Research Institute	•	
	Replication defective Simian adenovirus (GRAd) encoding S	Univercells/ReiThera/LEUKOCARE	•	
	ChAdOx1-S	AstraZeneca/Oxford University	•	
Inactivated	Inactivated	Chinese Academy of Medical Sciences	•	
		Sinovac	•	
		Sinopharm/Wuhan Institute of Biological Products	•	
	Whole-Virion Inactivated	Bharat Biotech	•	
DNA	Plasmid vaccine with electroporation	International Vaccine Institute/Inovio Pharmaceuticals		•
	Plasmid vaccine + Adjuvant	AnGes/Takara Bio/Osaka University	•	
	Plasmid vaccine	Cadila Healthcare Limited		•
	Vaccine (GX-19)	Genexine Consortium	•	
Virus-like particles	Plant-derived VLP adjuvanted with GSK or Dynavax adjs.	GSK	•	
Replicating viral vector	Measles-vector based	Pittsburg University CVR/Merck Sharp & Dohme/Institute Pasteur/Themis	•	
		Sinopharm/Beijing Institute of Biological Products	•	
Here, • = Yes; Blank = No				

Another vaccine at the clinical trial level is S gene-based synthetic vaccine, INO-4800 (NCT04336410). It is a DNA plasmid vaccine with electroporation for intradermal route of administration. This open-label study to evaluate the immunogenicity, tolerability, and safety profile of INO-4800 vaccine by testing two different dosages using CELLECTRA^®^ 2000. It is currently in phase 1 (n=120) of the clinical trial with a time frame of 28 weeks, developed by Inovio Pharmaceuticals in Missouri and Pennsylvania, and manufactured by International Vaccine Institute, United States.

Two inactivated vaccine types are being evaluated for the safety and immunogenicity for SARS-CoV-2. Both are randomized, placebo-controlled, and double-blind evaluation studies. One sponsored by Sinovac Biotech Co., Ltd, at Jiangsu, China (NCT04352608) (n=744 estimated) is to evaluate the safety indices of adverse reactions (0–28 days), immunogenicity indexes of neutralizing-antibody (emergency vaccination schedule; 0–14 days, nonemergency vaccination schedule; 0–28 days) for 2 doses. The second is Vero cells based inactivated vaccine (ChiCTR2000031809), sponsored by Henan provincial center for disease control and prevention, China to be conducted in Beijing Institute of Biological Sciences/Wuhan Institute of Biological Sciences for immunogenicity and safety.

The fifth probable vaccine is mRNA-1273 (NCT04283461), which is under open-label dose-ranging trial (n=45 estimated) to evaluate its immunogenicity, reactogenicity, and safety upon twice 0.5 ml IM dose administration to deltoid muscle on 1st and 29th day. mRNA-1273 encodes for a full-length, prefusion stabilized S protein of SARS-CoV-2 and is a novel lipid nanoparticle (LNP)-encapsulated mRNA-based vaccine sponsored by ModernaTX, Inc, United States.

ChAdOx1 (NCT04324606) is another probable vaccine being developed using nonreplicating vector technology to be tested with IM administration. It is based on the adenovirus vaccine vector with SARS-CoV-2 spike protein. Developed by the University of Oxford, United Kingdom, and to be manufactured by AstraZeneca, aimed to conduct a randomized, single-blinded, multi-center phase 1 and 2 study (n= 1112 estimated) to assess the immunogenicity, safety, and efficacy of the ChAdOx. It is single-dose probable vaccine.

There are some other notable probable vaccines are Anhui Zhifei Longcom Biopharmaceutical along and Institute of Microbiology, Chinese Academy of Sciences sponsored adjuvanted recombinant protein (RBD-Dimer), Osaka University, AnGes and Takara Bio sponsored DNA plasmid vaccine and adjuvant, Bharat Biotech’s whole virion inactivated vaccine, Cadila’s DNA plasmid vaccine, Arcturus/Duke-NUS sponsored mRNA vaccine, Genexine Consortium’s GX-19 DNA vaccine, etc.

Apart from above mentioned probable vaccines, Codagenix and Serum Institute of India are investing in viral deoptimization to synthesize rationally designed live attenuated vaccine. It is in the pre-clinical stage and technical details of the proposed vaccine are awaited. To date, all these vaccine proposals are in the trial phase, and there is no approved vaccine specific for SARS-CoV-2 antiviral activity. In the pandemic paradigm, the development of a vaccine needs a quick start with many parallel steps without a pause as compared to the traditional vaccine development where linear steps with multiple analysis pauses are taken to reduce the attrition rate and financial losses. Therefore, it is imperative that some of these may fail; however, chances of getting a vaccine specific to SARS-CoV-2 seems to be higher due to the vast understanding of the viral genome at an unprecedented speed.

## Conclusion

In the current outbreak, it is imperative to understand which patients might benefit from which set of therapy, especially when more than one disease progression pattern exists. The clinical data available so far is scant and inconsistent; therefore, a set of drugs or combinations or support therapy with complete therapeutic confidence is difficult to derive. Nevertheless, understanding of the treatment and management of COVID-19 patients is evolving very fast. The continuous emergence of clinical trial outcomes, pharmacologic therapy, and efficacy of available drugs for SARS-CoV-2 as clinical data is eagerly awaited. Investigation to assess the therapeutic promises of these drugs require a large number of controlled clinical trials and comparative evaluation for a better understanding of their effectiveness in COVID-19.

## Author Contributions

All authors contributed to the article and approved the submitted version.

## Conflict of Interest

The authors declare that the research was conducted in the absence of any commercial or financial relationships that could be construed as a potential conflict of interest.
